# Vesicular Communication in the Bone–Muscle Unit: Physiological Functions, Aging, and Therapeutic Potential

**DOI:** 10.3390/cells15151413

**Published:** 2026-08-04

**Authors:** Virginia Veronica Visconti, Chiara Greggi, Antonio Matticari, Riccardo Iundusi, Elena Gasbarra, Annalisa Botta, Umberto Tarantino

**Affiliations:** 1 Department of Biomedicine and Prevention, University of Rome “Tor Vergata”, 00133 Rome, Italy; virginia.veronica.visconti@uniroma2.it (V.V.V.); botta@med.uniroma2.it (A.B.); 2 Department of Orthopaedics and Traumatology, “Policlinico Tor Vergata” Foundation, Viale Oxford 81, 00133 Rome, Italy; antonio.matticari@gmail.com (A.M.); riccardo.iundusi@uniroma2.it (R.I.); elenagasbarra@tiscali.it (E.G.); umberto.tarantino@uniroma2.it (U.T.); 3 Department of Clinical Sciences and Translational Medicine, University of Rome Tor Vergata, Via Montpellier 1, 00133 Rome, Italy; 4 Faculty of Medicine and Surgery, University “Our Lady of Good Counsel”, Rruga Dritan Hoxha, 1000 Tirana, Albania

**Keywords:** extracellular vesicles (EVs), muscle, bone, bone–muscle crosstalk, osteoporosis, sarcopenia, musculoskeletal diseases

## Abstract

Extracellular vesicles (EVs) have emerged as fundamental pillars of intercellular communication, acting as primary mediators of the bidirectional biochemical crosstalk within the integrated bone–muscle unit. This review provides a comprehensive synthesis of EV-mediated signaling across the bone–muscle axis, offering a side-by-side mapping of vesicular biogenesis, cargo composition, and functional roles in both tissues. Under physiological conditions, skeletal muscle- and bone-derived EVs orchestrate tissue homeostasis, adaptations to physical exercise, myogenesis, and bone remodeling by transferring unique molecular cargos of proteins and specific microRNAs. However, aging induces a profound remodeling of the EV secretome toward a senescent profile characterized by harmful vesicular factors. This dysfunctional vesicular signaling impairs both muscle regeneration and osteogenesis, directly contributing to the pathogenesis of interconnected age-related disorders like sarcopenia, osteoporosis, and osteosarcopenia. Concurrently, circulating EVs represent valuable, minimally invasive biomarkers for early diagnosis. On the therapeutic front, this review critically evaluates emerging EV-based approaches, utilizing mesenchymal stem cell-derived, bioengineered, or biomaterial-incorporated EVs, offering promising, low-immunogenic alternatives to cell transplantation to enhance musculoskeletal tissue repair and restore bone–muscle homeostasis. Despite persisting technical challenges regarding large-scale production and standardization, targeting or leveraging EV-mediated communication represents one of the most innovative and revolutionary strategies to counteract age-related musculoskeletal decline. By unifying physiological mechanisms, age-related molecular reprogramming, and therapeutic engineering across both muscle and bone into a single narrative, this review provides a comprehensive framework to guide future research and clinical translation in musculoskeletal health.

## 1. Introduction

Extracellular vesicles (EVs), defined as nano- to micro-sized, lipid bilayer-delimited particles naturally released by all cell types that cannot replicate, have become a fundamental pillar in the study of intercellular and tissue communication, overcoming the old paradigm that considered them mere cellular waste [1]. EV biogenesis is a highly orchestrated process that allows for their classification mainly into two major populations: exosomes and microvesicles. In this context, a historical milestone in the field was the pioneering identification of matrix vesicles in 1967 by Bonucci and Anderson. These specialized membrane-bound structures, responsible for initiating extracellular matrix mineralization, represented one of the earliest demonstrations that cell-released vesicles execute primary physiological roles. Today, we know that microvesicles form by direct budding of the plasma membrane, whereas exosomes have an endosomal origin; indeed, they are generated within multivesicular bodies which, by fusing with the cell membrane, release the exosomes into the extracellular space [2,3,4]. Once released, EVs carry a specific molecular cargo comprising miRNAs, proteins, lipids, and nucleic acids capable of faithfully reflecting the pathophysiological state of the cell of origin. Recently, this signaling system has been identified as the primary mediator of the bidirectional crosstalk between skeletal muscle and bone tissue. During processes such as physical exercise or aging, both bone- and muscle-derived EVs are actively programmed in response to mechanical and biochemical stimuli: myocytes release EVs loaded with myogenic factors and specific miRNAs that are taken up by osteoblasts to stimulate new bone deposition, while osteocytes and osteoblasts respond by secreting EVs that transfer regulatory signals to muscle satellite cells, coordinating fiber growth and contractility [5,6].

The alteration of this delicate biological balance plays a key role in the pathophysiology of musculoskeletal aging, which often culminates in two closely interconnected conditions: sarcopenia, a geriatric syndrome characterized by the progressive and generalized loss of skeletal muscle mass, strength, and function, and osteoporosis, a systemic skeletal disease distinguished by reduced bone mineral density and microarchitectural deterioration of the tissue, leading to increased bone fragility and fracture risk. It has been demonstrated that dysfunctional EVs loaded with specific pro-inflammatory and pro-fibrotic miRNAs directly contribute to the pathogenesis of sarcopenia—by blocking myoblast differentiation and muscle regeneration—and of osteoporosis—by unbalancing bone remodeling in favor of osteoclastic resorption [7]. Precisely because of this capacity to reflect the health status of their tissues of origin, circulating EVs are emerging not only as promising, minimally invasive molecular markers for the early diagnosis of these diseases, but also as innovative biological therapeutic vectors, capable of transferring regenerative molecules to counteract muscle atrophy and the loss of bone mineral density [5,7].

The present review provides a systematic and symmetrical synthesis of extracellular vesicle (EV) signaling across the bone–muscle axis. Building upon current knowledge in musculoskeletal signaling, this work delivers a parallel, side-by-side mapping of EV biogenesis, specific miRNA cargos, and functional mechanisms in both skeletal muscle and bone tissue. By structured comparison, it illustrates how physiological vesicular homeostasis shifts toward a senescent secretome in both compartments during aging, while critically evaluating how emerging bioengineered EVs and biomaterial-based delivery platforms can be leveraged to restore bone–muscle balance.

## 2. Skeletal Muscle-Derived Extracellular Vesicles: Cargo Composition and Roles in Local and Systemic Communication

Skeletal muscle, which accounts for approximately 40% of total body mass and serves as the body’s main protein reservoir, is now widely recognized as an endocrine organ [8]. Communication between skeletal muscle and other tissues was initially attributed to the release of myokines and exerkines, which play autocrine, paracrine, and endocrine effects [9]. More recently, the concept of the muscle secretome has expanded to include EVs, now considered key mediators of intercellular communication [10,11]. Myoblasts and myotubes release EVs containing selectively packaged proteins, RNAs, and miRNAs, involved in essential biological processes, including signal transduction, transcriptional regulation, ion transport, and membrane function [12]. In particular, pioneering isolation of microvesicles released by differentiating C2C12 myoblasts demonstrated that muscle-derived EVs directly transport muscle-specific microRNAs (such as miR-133a) alongside full-length mitochondrial DNA (mtDNA) transcripts, establishing that myogenic cells utilize vesicular secretion to export both functional genetic materials and bioenergetic signals into the extracellular environment [13]. Furthermore, EVs can transfer their cargo between muscle cells, influencing myogenesis and differentiation, thereby contributing to the maintenance of skeletal muscle homeostasis [14]. Physical exercise is one of the main physiological stimuli capable of modulating EV release [15]. Several studies have shown that a single bout of aerobic exercise, as well as endurance or high-intensity training, rapidly increases circulating EV levels, suggesting an acute muscle response to contractile stress [5]. In human validation experiments during cycling exercise, a single bout of exhaustive aerobic exertion was shown to induce a rapid, temporary elevation of circulating small EVs carrying classical exosomal markers CD63, CD81, and TSG101, which returned to baseline levels within 90 min post-exercise, indicating an immediate cellular stress response and rapid systemic clearance mechanism [16]. Despite these findings, the actual contribution of skeletal muscle to the circulating EVs pool has long remained controversial. Available evidence indicates that a substantial proportion of EVs released by skeletal muscle remains within the muscle interstitial compartment, where they can directly interact with neighboring myofibers, satellite cells, endothelial cells, and resident immune cells [17,18]. Within this microenvironment, EVs can be rapidly internalized through endocytosis or receptor-mediated uptake, contributing to the regulation of local processes such as extracellular matrix remodeling, transient inflammatory responses, and exercise-induced metabolic adaptations [19].

Nevertheless, beyond their predominantly local role, skeletal muscle-derived EVs can also contribute to systemic inter-organ communication, albeit to a lesser extent. A subset of these vesicles enters the bloodstream and acts as a carrier of diverse bioactive molecules [6]. Through this mechanism, muscle-derived EVs function as biological messengers capable of influencing multiple tissues. In adipose tissue, they regulate adipocyte development and lipid metabolism, balancing lipid storage and utilization while promoting white adipose tissue browning [20,21]. In the liver, muscle-derived EVs modulate glucose production and utilization, thereby influencing whole-body glucose and lipid homeostasis through the regulation of key metabolic pathways and enzymes [22]. In the pancreas, they regulate β-cell function, directly affecting both insulin secretion and the tissue’s ability to respond appropriately to changes in blood glucose levels [23]. Their effects on the cardiovascular system include the maintenance of vascular health and the promotion of angiogenesis through the delivery of factors that support blood vessel growth and circulation [24]. Muscle-derived EVs also influence the central nervous system by modulating neurogenesis, reducing neuroinflammatory processes, and regulating neuronal signaling and plasticity [25]. Furthermore, they play an important role in immune regulation by influencing macrophage activation and fine-tuning the overall inflammatory response [26].

In addition to their direct biological effects, EVs can serve as carriers for numerous myokines, enhancing their stability and bioavailability in peripheral tissues [11]. These include interleukin-6 (IL-6), involved in energy metabolism and immune regulation; IL-8, which promotes angiogenesis; IL-15, associated with muscle growth and reduced adiposity; brain-derived neurotrophic factor (BDNF), implicated in neuronal function and energy metabolism; and irisin, which stimulates the browning of white adipose tissue and may also contribute to bone remodeling. EVs can also transport members of the TGF-β family, including myostatin, a potent negative regulator of muscle growth and myogenesis [11] (Table 1).

## 3. Physiological Functions of EVs Within Skeletal Muscle

Within muscle tissue, EVs represent an important local communication system that substantially contributes to the fine-tuning of tissue homeostasis. Through the selective transfer of miRNAs, proteins, lipids, metabolic enzymes, and regulatory transcripts, EVs dynamically modulate key processes such as myogenesis, muscle regeneration, and functional adaptation to contractile stimuli, acting as coordinating mediators between muscle cells and the microenvironment [49]. During myogenesis, EVs contribute to regulating the balance between proliferation and differentiation of myogenic progenitor cells. Indeed, muscle EVs contain a highly conserved set of myomiRNAs, including miR-1, miR-133a/b, and miR-206, which represent fundamental regulatory hubs of the myogenic program [7]. Specifically, miR-1 and miR-206 promote the activation of the myogenic differentiation program by modulating specific transcription factors, whereas miR-133 favors myoblast proliferation, contributing to the maintenance of the progenitor pool [18]. In addition to miRNAs, EVs transport proteins involved in cytoskeletal regulation, such as actin, tubulin, and annexins (Annexin A1/A2), as well as proteins associated with signal transduction and stress response, including Heat Shock Protein 70 (HSP70) and 14-3-3 proteins, suggesting a coordinated role in the progression of the differentiation program [12].

Following tissue damage, EVs modulate muscle regeneration, mediating communication among damaged muscle fibers, satellite cells, and stromal cells of the connective tissue, orchestrating the phases of myoblast activation, proliferation, and fusion. Furthermore, myogenic cell-derived EVs contain miRNAs and regulatory proteins capable of modulating the expression of genes involved in cell proliferation, local inflammation, and extracellular matrix remodeling. Among these, miR-1, miR-133a, miR-133b, miR-206, miR-486, and miR-181a contribute to the regulation of muscle growth, differentiation, and regeneration processes [27]. In particular, it has been demonstrated that specific miRNAs, including miR-29a/b/c, miR-133a/b, miR-1, and miR-206, can influence fibroblast activity by regulating the expression of genes involved in collagen synthesis and extracellular matrix remodeling. Through the modulation of pro-fibrotic pathways, including TGF-β-mediated signaling, these miRNAs help maintain the balance between regeneration and fibrosis, an essential mechanism to ensure effective muscle tissue repair without excessive extracellular matrix deposition [50].

Physical exercise constitutes one of the main physiological stimuli capable of modulating biogenesis and release of muscle EVs. Exercise also modifies the molecular cargo of EVs, with variations depending on the intensity and duration of the effort, indicating that the muscle actively “programs” the vesicle content based on the type of stimulus [51]. Indeed, during training-induced hypertrophy processes, myogenic progenitor cells release EVs containing miR-206, which can be transferred to fibroblasts, downregulating the expression of Ribosome Binding Protein 1 (Rrbp1), a key factor in collagen synthesis. This mechanism contributes to limiting excessive extracellular matrix deposition and ensuring a physiological remodeling of muscle tissue in response to mechanical load [28]. In the absence of adequate satellite cell function, this balance is disrupted, favoring fibrotic processes and tissue dysfunction. Moreover, EVs derived from exercising muscle are also enriched in molecules involved in the regulation of glucose and lipid metabolism, the response to oxidative stress, and the structural remodeling of muscle fibers [5] (Table 1).

## 4. Aging and Pathological Remodeling of Muscle EVs

**Figure 1:**
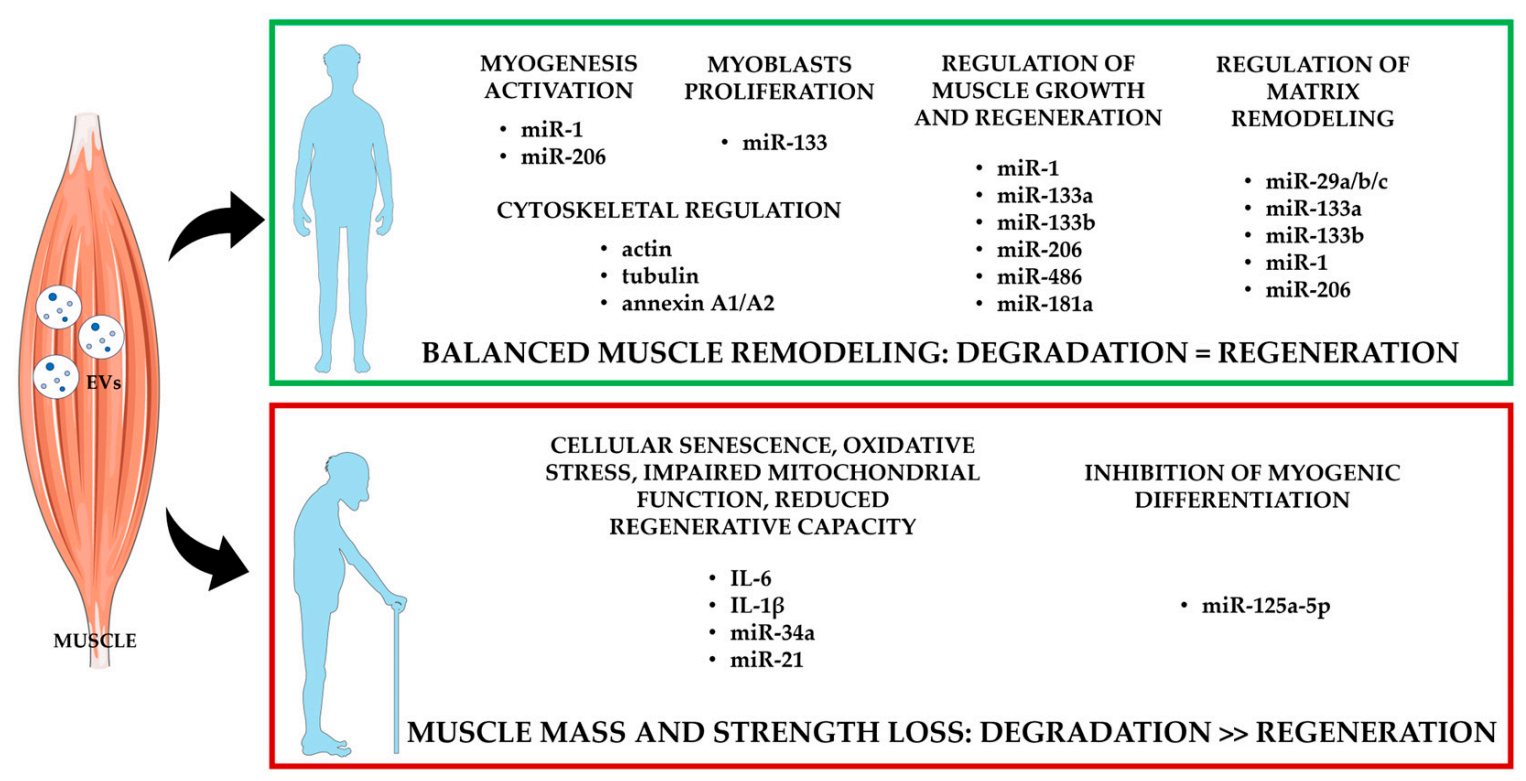
**Factors released by EVs in healthy and aging skeletal muscle.** In healthy skeletal muscle, the release of EVs promotes myogenesis, myoblast proliferation, and cytoskeletal and extracellular matrix regulation, thereby maintaining the homeostatic balance between muscle degradation and regeneration. During aging, the molecular profile shifts toward cellular senescence, oxidative stress, and inhibition of myogenic differentiation. Pro-inflammatory cytokines and specific microRNAs contribute to a marked predominance of muscle degradation over regeneration.

Aging is associated with a profound remodeling of the secretome of skeletal muscle-derived EVs, a phenomenon that significantly contributes to the pathophysiology of sarcopenia. In this context, EVs become not only vehicles for intercellular communication but also biological indicators of muscle decline and potential mediators of the processes driving the progression of sarcopenia and physical frailty [52,53]. One of the most significant changes concerns the acquisition of a secretory profile associated with cellular senescence. EVs released by aged muscle are enriched in components of the senescence-associated secretory phenotype (SASP), including pro-inflammatory cytokines such as IL-6 and IL-1β, molecules involved in extracellular matrix remodeling, and senescence-associated miRNAs, including miR-34a and miR-21. In particular, Fulzele and colleagues demonstrated that miR-34a significantly increases in muscle EVs with age and that its transfer to bone marrow stromal cells induces senescent phenotypes, promoting oxidative stress, alterations in mitochondrial function, and a reduction in regenerative capacity [29]. The sarcopenic state is accompanied by a remodeling of the content of muscle-specific and regeneration-regulating miRNAs. Indeed, under conditions of age-related atrophy, skeletal muscle-derived EVs display enrichment in miRNAs capable of suppressing myogenic programs and promoting muscle mass loss. In particular, it has been demonstrated that muscle EVs containing miR-125a-5p inhibit myogenic differentiation and bone formation through the modulation of pathways linked to cell growth and tissue remodeling, thereby contributing to the osteosarcopenic phenotype of aging [30]. In addition, aging more generally compromises the capacity of muscle EVs to sustain efficient and coordinated intercellular communication within the tissue. Indeed, EVs derived from aged muscle display a reduced functional capacity to maintain proper dialogue among muscle fibers, satellite cells and the stromal compartment, with direct consequences on the quality of tissue regeneration [29]. Under physiological conditions, EVs contribute to the activation of satellite cells through the transfer of miRNAs and protein signals that modulate key pathways such as MyoD, Myf5, and Pax7, as well as finely regulating the transition between proliferation and differentiation. In aging, this capacity is reduced, partly due to an alteration in vesicular cargo, which becomes depleted of pro-myogenic signals and instead enriched in molecules that promote cell cycle arrest and senescence [52] (Figure 1) (Table 1).

## 5. Bone-Derived EVs: Cargo Composition and Role in Skeletal Homeostasis

EVs are key mediators of skeletal homeostasis, facilitating local and systemic intercellular communication within the bone microenvironment [54,55]. Their cargo includes proteins, lipids, mRNAs, and miRNAs that regulate osteogenesis, osteoclastogenesis, and bone remodeling [34,56]. Among these, osteogenic miRNAs such as miR-29a/b, miR-218, miR-196a, and miR-335-5p promote osteoblast differentiation and matrix production by enhancing the expression of key osteogenic transcription factors, including RUNX2 and Osterix [34,35].

Bone-derived EVs also transport regulators of major signaling pathways involved in skeletal metabolism, including Wnt/β-catenin, Bone Morphogenetic Proteins (BMPs), TGF-β, and RANKL signaling [43]. These molecules stimulate osteogenic commitment of mesenchymal stem cells, regulate extracellular matrix synthesis and mineralization, and contribute to the coupling of bone formation and resorption [56,57]. In particular, EV-associated RANKL and osteoprotegerin (OPG) help control osteoclast differentiation and activity, thereby maintaining bone remodeling balance.

EVs mediate communication among osteoblasts, osteocytes, and osteoclasts, coordinating cellular responses required for skeletal maintenance [43]. Osteoblast-derived EVs promote osteogenic differentiation and mineralization through the transfer of pro-osteogenic proteins and miRNAs, whereas osteoclast-derived EVs deliver regulatory molecules, including miR-214, that modulate osteoblast activity and contribute to the coupling of bone resorption and formation [58,59,60]. Osteocytes, the primary mechanosensory bone cells, release EVs in response to mechanical stimulation, transmitting signals that regulate bone adaptation to loading and preserve skeletal integrity. In this context, mechanically induced Ca^2+^ oscillations in osteocytes trigger the release of EVs that significantly enhance bone formation [61]. Crucial to this process is the vesicular cargo of specific microRNAs, such as miR-21 and miR-195a-5p, which selectively target osteoblasts and osteoclasts to orchestrate bone formation and inhibit bone resorption [36] (Table 1). More recently, primary experimental studies have further expanded the role of EV cargo in skeletal regeneration. EVs released by H_2_S-stimulated M2 macrophages were shown to enhance the osteogenic differentiation of mesenchymal stem cells through enrichment of the membrane-associated protein moesin and activation of Wnt/β-catenin signaling, significantly improving bone regeneration in a murine calvarial defect model [62]. These findings reinforce the concept that the biological activity of EVs depends not only on their cellular origin but also on dynamic changes in their molecular cargo induced by mechanical, metabolic, and microenvironmental stimuli, thereby expanding their potential as therapeutic agents for bone regeneration and osteoporosis.

## 6. Physiological Functions of EVs in Bone Remodeling

EVs play a central role in the regulation of physiological bone remodeling by coordinating cellular responses involved in skeletal renewal, adaptation, and repair [43]. Bone remodeling is a tightly regulated process requiring the coordinated activity of osteoblasts, osteoclasts, osteocytes, and mesenchymal stem cells, and EV-mediated communication has emerged as a crucial mechanism underlying this cellular coordination [63,64].

At the cellular level, bone- and muscle-derived EVs exert their biological activities through distinct autocrine, paracrine, and juxtacrine modes of action [65]. In autocrine mechanisms, progenitor cells or osteoblasts internalize self-released EVs to sustain lineage commitment and intrinsic metabolic activity. In paracrine signaling, EVs diffuse through the local interstitial fluid to orchestrate reciprocal communication among adjacent bone cells, acting as vital coupling factors that coordinate bone resorption with subsequent formation. Furthermore, EVs execute juxtacrine functions through direct surface ligand–receptor interactions, where membrane-bound molecules (such as vesicle-anchored RANKL or Ephrin ligands) physically trigger intracellular signaling in opposing target cells prior to or independent of full vesicle endocytosis [65,66]. Through these multifaceted cellular pathways, EV-encapsulated myomiRs, coupling factors, and signaling proteins finely tune local cellular responses and skeletal homeostasis [65]. During osteogenesis, EVs released by osteoblasts and bone marrow-derived mesenchymal stem cells stimulate the expression of key osteogenic transcription factors, including RUNX2, Osterix (SP7), and activating transcription factor 4 (ATF4), as well as key functional enzymes such as alkaline phosphatase (ALP), thereby promoting lineage commitment and matrix deposition [56]. These effects are mediated by the transfer of osteogenic miRNAs, growth factors, and signaling molecules that activate Wnt/β-catenin and BMP signaling pathways in recipient cells [67]. Consequently, mesenchymal stem cells undergo differentiation toward mature osteoblasts capable of producing mineralized extracellular matrix [68].

In addition to regulating cellular differentiation, EVs directly contribute to extracellular matrix mineralization. EV membranes are enriched in phosphatidylserine, annexins, ALP, and calcium-binding proteins that create a microenvironment favorable for calcium and phosphate accumulation [46]. These vesicles serve as nucleation sites for hydroxyapatite crystal formation and growth, thereby initiating mineral deposition within the collagen-rich extracellular matrix. This function is particularly evident in matrix vesicles, specialized EVs secreted by osteoblasts and hypertrophic chondrocytes that represent the earliest sites of mineral crystal formation during skeletal development and fracture repair [69]. The fundamental concept of vesicular bone mineralization was historically established through ultrastructural electron microscopy, which revealed that 30–100 nm membrane-bounded matrix vesicles, budding from the plasma membrane of hypertrophic chondrocytes, serve as the initial site of apatite crystal deposition within the extracellular cartilage matrix prior to longitudinal bone formation [70].

Bone-derived EVs also play an essential role in the regulation of osteoclastogenesis and bone resorption [58]. Osteoblast- and osteocyte-derived EVs contain RANKL, a critical mediator of osteoclast differentiation and activation [44]. Following binding to RANK on osteoclast precursors, RANKL triggers intracellular signaling cascades involving TRAF6, NF-κB, MAPK, and NFATc1, leading to the formation of mature multinucleated osteoclasts capable of bone resorption [45]. Through the controlled transfer of RANKL and other regulatory molecules, EVs contribute to the fine-tuning of osteoclast activity according to local metabolic, hormonal, and mechanical requirements. Furthermore, osteoblast-derived microvesicles facilitate a crucial reciprocal crosstalk between osteoblasts and osteoclasts to balance bone remodeling [66]. Osteoclast-derived EVs have also emerged as important mediators of bone remodeling by transferring proteins and miRNAs, such as miR-214-3p, that inhibit osteoblast differentiation and contribute to the coupling between bone resorption and formation [37]. In addition, EVs modulate the RANKL/TRAF6/NF-κB/MAPK signaling pathway, thereby regulating osteoclast differentiation and resorptive activity [62]. Since chronic activation of NF-κB/MAPK is a common feature of both osteoporosis and sarcopenia, osteoclast-derived EVs have been proposed as potential mediators of bone–muscle crosstalk, although direct experimental evidence in osteosarcopenia is still limited.

Moreover, osteoclast-derived EVs participate in feedback mechanisms that regulate osteoblast function. In particular, EVs enriched in miR-214 have been shown to suppress osteoblast differentiation and reduce bone formation by targeting transcriptional regulators involved in osteogenesis [37]. This feedback loop may serve as a physiological mechanism for coordinating bone formation with ongoing resorption, thereby preventing excessive skeletal turnover and preserving bone architecture.

Collectively, these findings demonstrate that EVs function as essential mediators of intercellular crosstalk within the bone microenvironment, integrating anabolic and catabolic signals to maintain bone mass, structural integrity, and mechanical competence throughout life (Table 1).

## 7. Aging and Pathological Remodeling of Bone EVs

Aging profoundly alters the composition and biological activity of bone-derived EVs, contributing to the progressive decline in skeletal homeostasis observed in elderly individuals [71]. Age-associated changes in EV cargo include the accumulation of pro-inflammatory cytokines, senescence-associated miRNAs, and oxidative stress-related molecules, which collectively impair osteogenic differentiation and promote osteoclastogenesis [72,73,74]. As a result, the balance between bone formation and resorption shifts toward net bone loss, contributing to the development of osteoporosis and other age-related skeletal disorders [75].

Among the miRNAs enriched in aged bone-derived EVs, miR-34a and miR-214 are particularly relevant [29,76]. Increased levels of miR-34a released by senescent Bone Marrow Mesenchymal Stem Cells (BMSCs) and senescent bone cells have been associated with impaired osteoblast differentiation and enhanced cellular senescence through the inhibition of pro-survival and osteogenic pathways, including SIRT1-mediated signaling [29]. Similarly, osteoclast-derived EVs enriched in miR-214 suppress osteoblast activity by targeting osteogenic regulators such as ATF4, thereby reducing bone formation and contributing to remodelling imbalance [38,39]. In line with this, osteoclast-derived microRNA-containing exosomes can selectively inhibit osteoblast activity and impair bone formation [77]. Interestingly, under specific physiological conditions or apoptotic processes, mature osteoclast-derived apoptotic bodies can conversely promote osteogenic differentiation via RANKL-mediated reverse signaling [78]. Other age-associated miRNAs, including miR-21 and miR-188, also participate in skeletal aging [40,41]. While miR-21 can modulate inflammatory and osteogenic responses depending on the cellular context, miR-188 promotes the age-related shift of mesenchymal stem cells from osteogenic toward adipogenic differentiation, leading to increased marrow adiposity and reduced bone regeneration [40,41,42].

In parallel, senescent bone cells release EVs enriched in components of the SASP, including pro-inflammatory cytokines such as TNF-α, IL-1β, and IL-6 [47]. These mediators stimulate osteoclast differentiation, inhibit osteoblast function, and reinforce chronic low-grade inflammation within the bone microenvironment [48]. Many of these effects are mediated through activation of the NF-κB signaling pathway, a central regulator of inflammaging that promotes the expression of genes involved in inflammation, bone resorption, and cellular senescence. Ultimately, pathological alterations in EV signaling can tip the homeostasis balance in vivo, as osteoblast-derived vesicles have been demonstrated to induce a functional switch from bone formation to bone resorption [79]. Aging is also associated with increased oxidative stress and accumulation of reactive oxygen species (ROS), which further impair osteoblast differentiation and enhance osteoclast activity [80]. In response to oxidative damage, activation of the p53/p21 pathway induces cellular senescence and alters EV secretion, generating a feed-forward cycle that propagates senescence signals to neighbouring cells [81].

**Figure 2:**
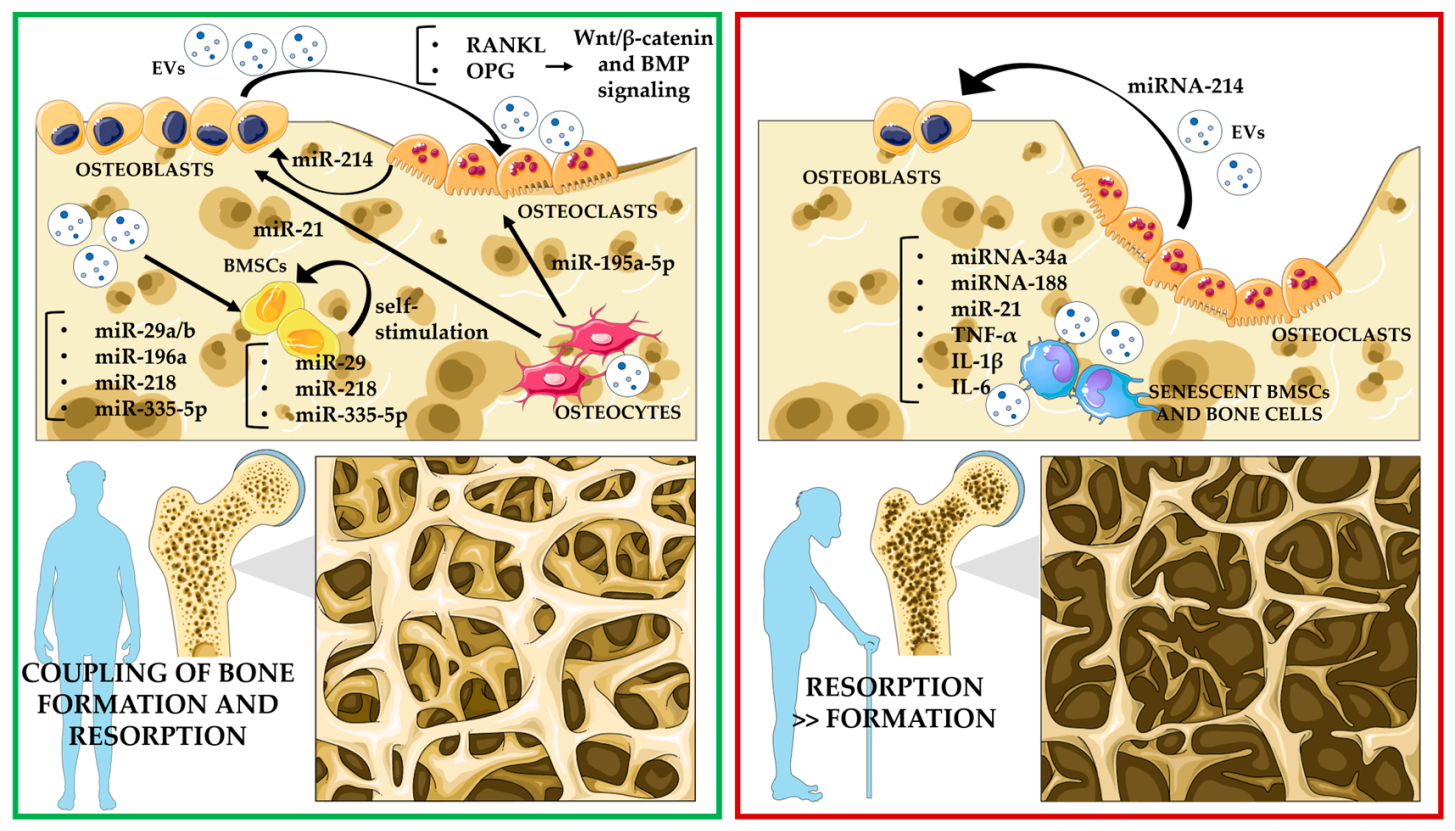
**EV-mediated intercellular communication mechanisms in physiological and pathological bone remodeling.** In a physiological context, there is a balanced coupling between bone formation and resorption, supported by a diverse cargo of microRNAs within EVs derived from osteoblasts, osteocytes, and BMSCs. The molecules released by these different bone and stromal cells cooperate to maintain the integrity and density of the trabecular structure. In aging, this balance is altered, leading to a clear prevalence of bone resorption over formation. This phenomenon, closely linked to cellular senescence processes, involves a shift in the profile of miRNAs and secreted factors (including pro-inflammatory cytokines). This leads to a progressive rarefaction of the bone texture and a loss of tissue mass.

Beyond their local effects on bone remodelling, age-related changes in EV cargo may influence distant tissues through systemic interorgan communication [82]. In particular, increasing evidence supports a role for bone-derived EVs in the crosstalk between bone and skeletal muscle. Dysregulation of this communication network may contribute to osteosarcopenia, a geriatric syndrome characterized by the coexistence of osteoporosis and sarcopenia [83]. Through the transfer of inflammatory mediators, senescence-associated miRNAs, and catabolic signaling molecules, aged EVs may simultaneously impair osteogenesis and muscle regeneration, highlighting their potential role as both biomarkers and therapeutic targets for age-related musculoskeletal decline (Figure 2) [84] (Table 1).

## 8. Bone–Muscle Crosstalk Mediated by EVs

Bone and skeletal muscle are increasingly recognized as components of an integrated musculoskeletal unit that communicates through both mechanical and biochemical signals. Importantly, while the direct dialogue between bone and muscle is central to musculoskeletal health, this axis operates within a broader systemic network. Emerging evidence highlights that inter-organ crosstalk involving other distant compartments, such as the gut–bone axis, also plays an important role in modulating tissue homeostasis, inflammation, and metabolic adaptations in the musculoskeletal system [85,86,87].

Beyond the classical actions of osteokines and myokines, EVs have recently emerged as important mediators of bone–muscle crosstalk [88]. Both tissues continuously release EVs into the local microenvironment and circulation, allowing the transfer of bioactive molecules capable of modulating cellular function in distant target organs. Evidence supporting EV-mediated communication between muscle and bone has accumulated in recent years. Skeletal muscle-derived EVs contain myomiRNAs, growth factors, cytokines, and metabolic regulators that can influence osteoblast differentiation and bone remodeling. Among these factors, irisin-associated signaling and several muscle-derived miRNAs have been implicated in the stimulation of osteogenic pathways and the maintenance of bone mass [31,32]. Specifically, in vivo administration of low-dose recombinant irisin in mice demonstrated a direct osteoanabolic effect, significantly increasing cortical bone mineral density, periosteal circumference, and polar moment of inertia by upregulating Runx2 and Atf4 expression in osteoblasts through p38 MAPK phosphorylation signaling pathways [33]. Conversely, bone-derived EVs can regulate muscle metabolism and regenerative capacity through the transfer of signaling molecules that influence myogenic differentiation, inflammation, and mitochondrial function [56,89]. The relevance of this communication becomes particularly evident during aging. Fulzele and colleagues demonstrated that aged skeletal muscle releases EVs enriched in miR-34a, which can be transferred to bone marrow stromal cells and induce cellular senescence, oxidative stress, and impaired regenerative capacity [29]. More recently, muscle-derived EVs containing miR-125a-5p have been shown to negatively regulate both myogenesis and osteogenesis, suggesting a potential mechanism linking sarcopenia and osteoporosis during aging [30]. These findings support the concept that age-related alterations in EV cargo contribute to the development of osteosarcopenia, a syndrome characterized by the simultaneous loss of muscle and bone mass.

Overall, EVs are increasingly recognized as fundamental components of the molecular dialogue between bone and muscle. By transporting regulatory miRNAs, proteins, and metabolites across tissues, EVs coordinate adaptive responses to mechanical loading, exercise, injury, and aging. Their involvement in both physiological and pathological musculoskeletal interactions highlights their potential as biomarkers of osteosarcopenia and as novel therapeutic targets aimed at preserving musculoskeletal health during aging [90]. The key molecular differences in EV cargo composition and their respective functional impacts between young and aged skeletal muscle and bone tissues are summarized in Table 2.

## 9. EVs as Diagnostic Biomarkers

Beyond their biological role in bone–muscle communication, EVs are increasingly recognized as promising liquid biopsy tools for age-related musculoskeletal disorders [32]. Because they are released into all biological fluids, including blood, urine, and saliva, EVs can be isolated through minimally invasive procedures while preserving the molecular signature of their cells of origin. Their cargo, including miRNAs, proteins, lipids, and other nucleic acids, changes according to physiological and pathological conditions, making EVs attractive candidates for disease diagnosis, prognosis, and therapeutic monitoring.

In the context of sarcopenia, osteoporosis, and osteosarcopenia, several studies have shown that alterations in circulating EVs concentration and cargo composition correlate with aging, chronic inflammation, impaired osteogenesis, and reduced muscle regenerative capacity [91]. In particular, age-associated EV cargo molecules such as miR-34a and miR-214 have been proposed as potential biomarkers of cellular senescence, impaired osteogenic differentiation, and muscle dysfunction [52]. Furthermore, experimental studies suggest that alterations in the systemic EVs profile may reflect the pathological bone–muscle communication underlying osteosarcopenia, supporting their potential use for early diagnosis, disease stratification, and monitoring of therapeutic responses [92]. Although the clinical application of EV-based biomarkers still requires standardized isolation methods and large-scale validation studies, their high stability in biological fluids and ability to provide real-time information on musculoskeletal homeostasis make them particularly attractive for precision medicine.

## 10. EVs as Therapeutic Tools

The growing understanding of EV-mediated communication within the musculoskeletal system has stimulated considerable interest in their therapeutic application for the treatment of sarcopenia, osteoporosis, and osteosarcopenia. Due to their intrinsic ability to transport bioactive molecules, low immunogenicity, and capacity to cross biological barriers, EVs are increasingly viewed as promising alternatives to conventional cell-based therapies [64]. In contrast to stem cell transplantation, EV-based approaches retain many of the beneficial paracrine effects of their parental cells while reducing concerns related to immune rejection, tumorigenicity, and poor cell engraftment [92].

Within skeletal muscle, EVs derived from MSCs, satellite cells, and young muscle tissue have shown the ability to promote myogenesis, enhance muscle regeneration, and attenuate inflammation in preclinical models of muscle injury and age-related muscle wasting [27]. These beneficial effects are largely mediated by the transfer of pro-regenerative miRNAs, growth factors, and anti-inflammatory molecules capable of stimulating satellite cell activation and improving the regenerative microenvironment. Similarly, in bone tissue, MSC-derived EVs have demonstrated significant osteoanabolic properties, promoting osteoblast differentiation, enhancing fracture healing, stimulating angiogenesis, and suppressing excessive osteoclast activity [31]. Recent advances in EV bioengineering have further enhanced these regenerative properties. Engineered MSC-derived EVs enriched with osteogenic miRNAs, such as miR-20a, have been shown to promote osteogenic differentiation by targeting BAMBI and activating osteogenic signaling pathways, resulting in improved bone regeneration in osteoporotic animal models [57,93]. Likewise, hypoxia-preconditioned MSC-derived EVs enriched in miR-126 significantly enhanced angiogenesis and accelerated bone repair through activation of the Ras/ERK signaling pathway [94]. Surface-functionalized EVs carrying bone-targeting peptides have also demonstrated improved biodistribution and therapeutic efficacy by increasing selective accumulation within skeletal tissue [71]. More recently, engineering strategies have expanded beyond miRNA loading to include the delivery of therapeutic mRNAs. In particular, engineered small extracellular vesicles simultaneously loaded with VEGF-A and BMP-2 mRNAs and incorporated into an injectable PEGylated hydrogel promoted coupled angiogenesis and osteogenesis, resulting in significantly enhanced bone regeneration compared with conventional EV-based approaches [95].

In parallel, EV-inspired nanoplatforms and biomimetic extracellular vesicles have emerged as promising alternatives to native EVs, offering improved cargo loading efficiency, scalable production, and greater flexibility for surface functionalization while preserving the biological activity of natural vesicles (Table 3) [96].

Recent studies have further demonstrated that advanced surface engineering and tissue-specific functionalization substantially improve EV homing to bone lesions, increasing local retention and therapeutic efficacy while reducing off-target biodistribution. These next-generation engineered EVs provide more efficient delivery of osteogenic molecules and represent an important step toward precision regenerative medicine for osteoporosis and other musculoskeletal disorders [97,98].

Several studies have further shown that engineered EVs enriched with osteogenic miRNAs or bone-targeting molecules can enhance bone regeneration in models of osteoporosis and critical-size bone defects [64].

Beyond their tissue-specific actions, EVs are emerging as potential modulators of bone–muscle crosstalk. Given their ability to circulate systemically and transfer molecular cargo between distant organs, EVs may represent an attractive strategy to simultaneously target both muscle and bone deterioration. Experimental studies suggest that restoring a youthful EV profile or selectively inhibiting senescence-associated vesicular signals could ameliorate the pathological communication that contributes to osteosarcopenia [52]. In particular, targeting age-associated EV cargo molecules such as miR-34a and miR-214 has been proposed as a potential strategy to reduce cellular senescence, improve osteogenic capacity, and preserve muscle regenerative potential [29].

**Figure 3:**
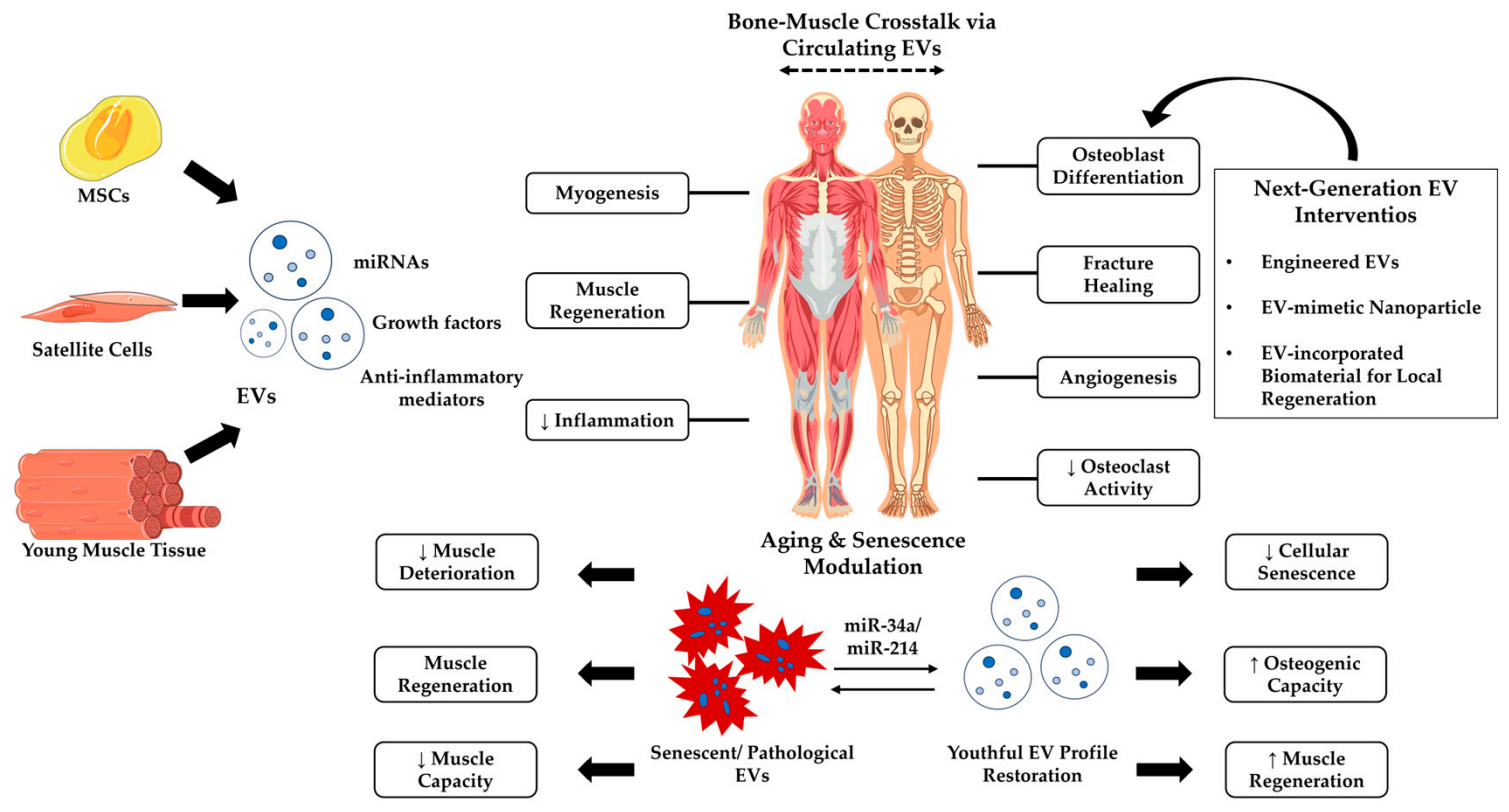
**Therapeutic applications of EVs in age-related musculoskeletal disorders.** EVs derived from MSCs, satellite cells, and young muscle tissue deliver bioactive cargo, including miRNAs, growth factors, and anti-inflammatory mediators, to promote myogenesis, muscle regeneration, and immune modulation. Through bone–muscle crosstalk, circulating EVs also enhance osteogenesis by stimulating osteoblast differentiation, angiogenesis, and fracture repair while inhibiting osteoclast activity. Restoration of a youthful EV profile, including modulation of miR-34a and miR-214, may counteract cellular senescence and improve musculoskeletal regenerative capacity.

Future therapeutic approaches may involve the development of bioengineered EVs loaded with specific miRNAs, proteins, or pharmacological agents, as well as the use of EV-mimetic nanoparticles designed to improve cargo delivery and tissue targeting. Furthermore, advances in tissue engineering have enabled the incorporation of EVs into biomaterials, hydrogels, and scaffold-based systems to enhance local musculoskeletal regeneration [99]. Although several technical challenges remain, including large-scale production, purification standardization, cargo heterogeneity, and biodistribution control, EV-based therapies represent one of the most promising emerging strategies for the treatment of age-related musculoskeletal disorders and the restoration of bone–muscle homeostasis (Figure 3) [92].

## 11. Conclusions

EVs represent an essential biological network that orchestrates the complex, bidirectional crosstalk between bone and skeletal muscle. As highlighted in this review, the transition from physiological homeostasis to age-related pathologies is heavily driven by the pathological remodeling of the EV secretome. The loss of pro-regenerative signals and the corresponding enrichment of senescence-associated cargos actively fuel the concurrent degradation of both tissues, establishing a key molecular mechanism underlying osteosarcopenia. Consequently, EVs hold dual clinical significance: they serve as sensitive, minimally invasive biomarkers for early musculoskeletal decline and offer a versatile platform for targeted therapies. While bioengineering strategies, tissue engineering scaffolds, and native EV-based interventions demonstrate substantial osteoanabolic and myogenic potential in preclinical models, translating these findings into clinical practice requires overcoming multiple biological, technological, manufacturing, and regulatory barriers [100].

One of the major translational challenges is achieving efficient and reproducible therapeutic cargo loading. Current endogenous and exogenous loading strategies frequently suffer from limited encapsulation efficiency, variable loading capacity, and the potential to alter EV membrane integrity or biological activity, thereby affecting therapeutic efficacy.

Another critical issue is tissue-specific targeting. Although surface engineering approaches based on targeting peptides, antibodies, aptamers, and other ligands have significantly improved EV homing in preclinical studies, selective delivery to bone and skeletal muscle following systemic administration remains suboptimal, limiting therapeutic precision. Standardizing purification protocols, scaling up large-scale production, reducing cargo heterogeneity, and achieving precise biodistribution control remain crucial hurdles. Moreover, the intrinsic heterogeneity of EV populations, including differences in cellular origin, size, membrane composition, molecular cargo, and biological function, represents one of the greatest obstacles to product characterization, quality control, potency assessment, and batch-to-batch reproducibility, thereby complicating regulatory approval and clinical translation [57].

Equally important is the establishment of robust Good Manufacturing Practice (GMP)-compliant manufacturing processes. Current isolation and purification methods, including ultracentrifugation, size-exclusion chromatography, tangential flow filtration, and affinity-based approaches, often require compromises between yield, purity, scalability, and production costs, highlighting the need for standardized, industrially scalable workflows.

Additional translational challenges include the development of optimized storage and formulation protocols. Freeze–thaw cycles, storage temperature, lyophilization procedures, and formulation buffers can significantly influence EV stability, cargo preservation, and biological activity, emphasizing the necessity for standardized preservation methods suitable for clinical use.

Long-term safety also requires careful evaluation. Although EVs generally exhibit lower immunogenicity and tumorigenic potential than cell-based therapies, comprehensive studies addressing pharmacokinetics, biodistribution, off-target accumulation, immunomodulatory effects, and the potential transfer of undesirable bioactive molecules remain essential before widespread clinical implementation.

Addressing these translational challenges will be pivotal to unlocking the full potential of EV-based nanomedicine and successfully restoring musculoskeletal health and quality of life in the aging population. Future progress will depend not only on advances in EV bioengineering and nanotechnology but also on the development of standardized potency assays, harmonized regulatory frameworks, and well-designed clinical trials capable of demonstrating the safety, reproducibility, and long-term efficacy of EV-based therapeutics in patients with age-related musculoskeletal disorders [100].

## Figures and Tables

**Table 1:** Representative protein and microRNA cargos of skeletal muscle- and bone-derived extracellular vesicles, their specific cellular origin, and biological roles.

Source Tissue	EV Cargo Component	Primary Biological Role/Target Mechanism	Physiological/ Pathological Context	References
Skeletal muscle	miR-1, miR-206	Activation of myogenesis and differentiation program	Muscle homeostasis, myogenesis	[7,18]
	miR-133a/b	Induction of myoblast proliferation and extracellular matrix regulation	Progenitor maintenance and regeneration	[18,27]
	miR-206	Downregulation of *Rrbp1* in fibroblasts to limit excessive collagen deposition	Exercise-induced remodeling	[28]
	miR-34a	BMSCs senescence induction; promotion of ROS release and oxidative stress	Musculoskeletal aging, sarcopenia	[29]
	miR-125a-5p	Suppression of myogenic differentiation and osteoblastogenesis impairment	Muscle atrophy, osteosarcopenia	[30]
	Annexins A1/A2, Actin Tubulin	Cytoskeletal organization and membrane dynamics	Myogenesis, cell differentiation	[12]
	HSP70 14-3-3 proteins	Stress response and intracellular signal transduction	Cellular stress adaptation	[12]
	Irisin, IL-6, IL-15, BDNF	Metabolic regulation, WAT browning, and osteoanabolic crosstalk	Systemic communication and crosstalk	[11,31,32,33]
Bone	miR-29a/b, miR-196a, miR-218, miR-335-5p	Upregulation of *RUNX2* and *Osterix* expression to stimulate osteoblast differentiation	Osteogenesis and bone formation	[34,35]
	miR-21, miR-195a-5p	Modulation of bone formation and inhibition of osteoclastic bone resorption	Mechanotransduction, homeostasis	[36]
	miR-214-3p	ATF4 silencing and bone formation impairment	Osteoclast-to-osteoblast inhibition	[37,38,39]
	miR-188	Shift in BMSCs fate from osteogenic to adipogenic	Bone marrow adiposity, bone aging	[40,41,42]
	RANKL, OPG	RANK binding on osteoclasts to trigger TRAF6/NF-κB signaling; bone resorption regulation	Osteoclastogenesis and remodeling	[43,44,45]
	ALP, Annexins	Provision of nucleation sites for hydroxyapatite crystal formation	Biomineralization	[46]
	SASP (TNF-α, IL-1β, IL-6)	Induction of chronic low-grade inflammation and bone resorption increment	Cellular senescence, osteoporosis	[47,48]

Abbreviations: ALP, Alkaline Phosphatase; ATF4, Activating Transcription Factor 4; BDNF, Brain-Derived Neurotrophic Factor; BMSCs, Bone Marrow Mesenchymal Stem Cells; HSP70, Heat Shock Protein 70; IL-1β/IL-6/IL-15, Interleukin-1β/6/15; miR/microRNA, OPG, Osteoprotegerin; RANKL, Receptor Activator of Nuclear Factor Kappa B; ROS, Reactive Oxygen Species; Rrbp1, Ribosome Binding Protein 1; SASP, Senescence-Associated Secretory Phenotype; TNF-α, Tumor Necrosis Factor-Alpha; WAT, White Adipose Tissue.

**Table 2:** Comparison of extracellular vesicle cargo in young and aged skeletal muscle and bone.

EV Origin	miRNA Cargo	Protein Cargo	Main Biological Effect
Young skeletal muscle	miR-1, miR-133a/b, miR-206	HSP70, Annexins, irisin, IL-15, BDNF	Promotes myogenesis, satellite cell activation, muscle regeneration, and supports osteogenesis.
Aged skeletal muscle	miR-34a, miR-125a-5p	IL-6, IL-1β, SASP-associated factors	Induces cellular senescence, impairs myogenesis and osteogenic support, and reduces regenerative capacity.
Young bone	miR-29a/b, miR-218, miR-196a, miR-335-5p, miR-21, miR-195a-5p	Balanced RANKL/OPG, ALP, Annexins	Promotes osteoblast differentiation, matrix mineralization, and physiological bone remodeling.
Aged bone	miR-214-3p, miR-188	TNF-α, IL-1β, IL-6 (SASP), altered RANKL/OPG	Enhances osteoclastogenesis, suppresses osteoblast activity, and promotes chronic inflammation and bone loss.

Abbreviations: ALP, alkaline phosphatase; BDNF, brain-derived neurotrophic factor; EV, extracellular vesicle; HSP70, heat shock protein 70; IL, interleukin; OPG, osteoprotegerin; RANKL, receptor activator of nuclear factor κB ligand; SASP, senescence-associated secretory phenotype; TNF-α, tumor necrosis factor-α.

**Table 3:** Current engineering strategies for EVs in musculoskeletal regenerative medicine.

Engineering Strategy	Engineering Approach	Therapeutic Cargo	Mechanism of Action	Musculoskeletal Application	References
Cargo loading with osteogenic miRNAs	Genetic engineering of parental MSCs or direct EV loading	miR-20a	Targets BAMBI and activates osteogenic signaling, promoting osteoblast differentiation and bone formation	Osteoporosis, bone regeneration	[93]
Hypoxia-preconditioned EVs	MSC culture under hypoxic conditions	miR-126-enriched EVs	Activates Ras/ERK signaling, enhances angiogenesis and accelerates bone repair	Osteoporosis, fracture healing	[94]
mRNA-engineered EVs	Active loading of therapeutic mRNAs into EVs	VEGF-A + BMP-2 mRNAs	Simultaneously promotes angiogenesis and osteogenesis, improving bone regeneration	Critical-size bone defects, osteoporosis	[95]
Surface-engineered EVs	Functionalization with bone-targeting peptides/ligands	Bone-targeting molecules	Improves bone homing, local retention, biodistribution and therapeutic efficacy	Osteoporosis, bone defects	[71,97,98]
Biomimetic EVs	Artificial or hybrid EV-like nanovesicles	Customizable therapeutic cargo	Increases cargo loading efficiency, scalability and stability while preserving EV bioactivity	Bone and skeletal muscle regeneration	[96]
EV-mimetic nanoparticles	Synthetic nanocarriers mimicking EVs	miRNAs, proteins, drugs	Improves targeted delivery and controlled release of therapeutic molecules	Osteoporosis, osteosarcopenia	[52]
Hydrogel-assisted EV delivery	Incorporation of EVs into injectable PEGylated hydrogels	Native or engineered EVs	Sustained local release, enhanced angiogenesis and osteogenesis	Bone regeneration	[95]
Scaffold-based EV delivery	Incorporation into biodegradable biomaterials/scaffolds	Native or engineered EVs	Improves local retention and creates a regenerative microenvironment	Bone defects, musculoskeletal regeneration	[99]
Native regenerative EV therapy	Administration of MSC-, satellite cell-, or young tissue-derived EVs	Endogenous miRNAs, growth factors, cytokines	Promotes myogenesis, osteogenesis, angiogenesis and reduces inflammation	Sarcopenia, osteoporosis, osteosarcopenia	[27,31,64]
Modulation of senescence-associated EV cargo	Targeting age-related EV miRNAs	miR-34a, miR-214	Reduces cellular senescence, restores bone–muscle crosstalk and regenerative potential	Osteosarcopenia	[29,52]

Abbreviations: BMP, Bone Morphogenetic Protein; ERK, Extracellular Signal-Regulated Kinase; EV, Extracellular Vesicle; GelMA, Gelatin Methacryloyl; MSCs, Mesenchymal Stem Cells; PEGylated, Polyethylene Glycol-modified; Ras, Rat Sarcoma Virus protein family; VEGF-A, Vascular Endothelial Growth Factor A.

## Data Availability

No new data were created or analyzed in this study. Data sharing is not applicable to this article.

## References

[1] Welsh J.A., Goberdhan D.C.I., O’DRiscoll L., Buzas E.I., Blenkiron C., Bussolati B., Cai H., Di Vizio D., Driedonks T.A.P., Erdbrügger U. (2024). Minimal information for studies of extracellular vesicles (MISEV2023): From basic to advanced approaches. J. Extracell. Vesicles.

[2] Théry C., Witwer K.W., Aikawa E., Alcaraz M.J., Anderson J.D., Andriantsitohaina R., Antoniou A., Arab T., Archer F., Atkin-Smith G.K. (2018). Minimal information for studies of extracellular vesicles 2018 (MISEV2018): A position statement of the International Society for Extracellular Vesicles and update of the MISEV2014 guidelines. J. Extracell. Vesicles.

[3] Bonucci E. (1967). Fine structure of early cartilage calcification. J. Ultrastruct. Res..

[4] Anderson H.C. (1967). Electron microscopic studies of induced cartilage development and calcification. J. Cell Biol..

[5] Whitham M., Parker B.L., Friedrichsen M., Hingst J.R., Hjorth M., Hughes W.E., Egan C.L., Cron L., Watt K.I., Kuchel R.P. (2018). Extracellular Vesicles Provide a Means for Tissue Crosstalk during Exercise. Cell Metab..

[6] Jia J., Wang L., Zhou Y., Zhang P., Chen X. (2025). Muscle-derived extracellular vesicles mediate crosstalk between skeletal muscle and other organs. Front. Physiol..

[7] Wang X., Huang H., Chen J., Zhang Q., Yuan Z., Yin M., Peng C., Liu S. (2026). Mechanism of exercise-derived circulating exosomes as a target for sarcopenia management. Front. Physiol..

[8] Pedersen B.K., Febbraio M.A. (2012). Muscles, exercise and obesity: Skeletal muscle as a secretory organ. Nat. Rev. Endocrinol..

[9] Chow L.S., Gerszten R.E., Taylor J.M., Pedersen B.K., van Praag H., Trappe S., Febbraio M.A., Galis Z.S., Gao Y., Haus J.M. (2022). Exerkines in health, resilience and disease. Nat. Rev. Endocrinol..

[10] Rome S. (2022). Muscle and Adipose Tissue Communicate with Extracellular Vesicles. Int. J. Mol. Sci..

[11] Trovato E., Di Felice V., Barone R. (2019). Extracellular Vesicles: Delivery Vehicles of Myokines. Front. Physiol..

[12] Forterre A., Jalabert A., Berger E., Baudet M., Chikh K., Errazuriz E., De Larichaudy J., Chanon S., Weiss-Gayet M., Hesse A.-M. (2014). Proteomic Analysis of C2C12 Myoblast and Myotube Exosome-Like Vesicles: A New Paradigm for Myoblast-Myotube Cross Talk?. PLoS ONE.

[13] Guescini M., Guidolin D., Vallorani L., Casadei L., Gioacchini A.M., Tibollo P., Battistelli M., Falcieri E., Battistin L., Agnati L.F. (2010). C2C12 myoblasts release micro-vesicles containing mtDNA and proteins involved in signal transduction. Exp. Cell Res..

[14] Rome S., Forterre A., Mizgier M.L., Bouzakri K. (2019). Skeletal Muscle-Released Extracellular Vesicles: State of the Art. Front. Physiol..

[15] Vechetti I.J., Valentino T., Mobley C.B., McCarthy J.J. (2020). The role of extracellular vesicles in skeletal muscle and systematic adaptation to exercise. J. Physiol..

[16] Frühbeis C., Helmig S., Tug S., Simon P., Krämer-Albers E. (2015). Physical exercise induces rapid release of small extracellular vesicles into the circulation. J. Extracell. Vesicles.

[17] Zheng Y., Streleckis A.C., Chen H., Yao Y. (2025). Enzymatic Isolation of Skeletal Muscle Interstitial Extracellular Vesicles. J. Vis. Exp..

[18] Watanabe S., Sudo Y., Makino T., Kimura S., Tomita K., Noguchi M., Sakurai H., Shimizu M., Takahashi Y., Sato R. (2022). Skeletal Muscle Releases Extracellular Vesicles with Distinct Protein and MicroRNA Signatures That Function in the Muscle Microenvironment. PNAS Nexus.

[19] Arif S., Moulin V.J. (2023). Extracellular vesicles on the move: Traversing the complex matrix of tissues. Eur. J. Cell Biol..

[20] Srivastava S., Rathor R., Singh S.N., Suryakumar G. (2021). Emerging role of MyomiRs as biomarkers and therapeutic targets in skeletal muscle diseases. Am. J. Physiol. Physiol..

[21] Price N.L., Fernández-Hernando C. (2016). miRNA regulation of white and brown adipose tissue differentiation and function. Biochim. Biophys. Acta.

[22] Rohm T.V., e Rocha K.C., Olefsky J.M. (2025). Metabolic Messengers: Small extracellular vesicles. Nat. Metab..

[23] Chidester S., Livinski A.A., Fish A.F., Joseph P.V. (2020). The Role of Extracellular Vesicles in β-Cell Function and Viability: A Scoping Review. Front. Endocrinol..

[24] Boulanger C.M., Loyer X., Rautou P.-E., Amabile N. (2017). Extracellular vesicles in coronary artery disease. Nat. Rev. Cardiol..

[25] Schnatz A., Müller C., Brahmer A., Krämer-Albers E. (2021). Extracellular Vesicles in neural cell interaction and CNS homeostasis. FASEB BioAdvances.

[26] Robbins P.D., Morelli A.E. (2014). Regulation of immune responses by extracellular vesicles. Nat. Rev. Immunol..

[27] Porcu C., Dobrowolny G., Scicchitano B.M. (2024). Exploring the Role of Extracellular Vesicles in Skeletal Muscle Regeneration. Int. J. Mol. Sci..

[28] Fry C.S., Kirby T.J., Kosmac K., McCarthy J.J., Peterson C.A. (2017). Myogenic Progenitor Cells Control Extracellular Matrix Production by Fibroblasts during Skeletal Muscle Hypertrophy. Cell Stem Cell.

[29] Fulzele S., Mendhe B., Khayrullin A., Johnson M., Kaiser H., Liu Y., Isales C.M., Hamrick M.W. (2019). Muscle-derived miR-34a increases with age in circulating extracellular vesicles and induces senescence of bone marrow stem cells. Aging.

[30] Shao X., Zhang P., Fan Z., Lin J., Chen X., Liu N., Gong W., He Y., Zhou Y., Shi T. (2026). Atrophic Skeletal Muscle-Derived Extracellular Vesicles Transfer miR-125a-5p to Inhibit Bone Formation in Osteoporosis during Aging. Adv. Sci..

[31] Qin W., Dallas S.L. (2019). Exosomes and Extracellular RNA in Muscle and Bone Aging and Crosstalk. Curr. Osteoporos. Rep..

[32] Fu P., Luo Y., Zheng S., Ma Z., Xiao W., Li H., Li Y. (2025). The Role of Extracellular Vesicles in Musculoskeletal Diseases. J. Extracell. Vesicles.

[33] Colaianni G., Cuscito C., Mongelli T., Pignataro P., Buccoliero C., Liu P., Lu P., Sartini L., Di Comite M., Mori G. (2015). The myokine irisin increases cortical bone mass. Proc. Natl. Acad. Sci. USA.

[34] Jing D., Hao J., Shen Y., Tang G., Li M.-L., Huang S.-H., Zhao Z.-H. (2015). The role of microRNAs in bone remodeling. Int. J. Oral Sci..

[35] Singh M., Singh P., Singh B., Sharma K., Kumar N., Singh D., Mastana S. (2024). Molecular Signaling Pathways and MicroRNAs in Bone Remodeling: A Narrative Review. Diseases.

[36] Yuze M., Hu J., Jun L., Cheng X., Tianwen X., Junqiang Z. (2025). Osteocytes function as biomechanical signaling hubs bridging mechanical stress sensing and systemic adaptation. Front. Physiol..

[37] Li D., Liu J., Guo B., Liang C., Dang L., Lu C., He X., Cheung H.Y.-S., Xu L., Lu C. (2016). Osteoclast-derived exosomal miR-214-3p inhibits osteoblastic bone formation. Nat. Commun..

[38] Safaei S., Sohrabi S., Zahmatkesh P., Soltani-Zangbar M.S., Maleki L.A. (2025). Exosomes in aging and age-related disorders: Mechanisms, therapeutic potentials, and challenges. J. Transl. Med..

[39] Fang F., Yang J., Wang J., Li T., Wang E., Zhang D., Liu X., Zhou C. (2024). The role and applications of extracellular vesicles in osteoporosis. Bone Res..

[40] Li C.-J., Cheng P., Liang M.-K., Chen Y.-S., Lu Q., Wang J.-Y., Xia Z.-Y., Zhou H.-D., Cao X., Xie H. (2015). MicroRNA-188 regulates age-related switch between osteoblast and adipocyte differentiation. J. Clin. Investig..

[41] Subramaniam R., Vijakumaran U., Shanmuganantha L., Law J.-X., Alias E., Ng M.-H. (2023). The Role and Mechanism of MicroRNA 21 in Osteogenesis: An Update. Int. J. Mol. Sci..

[42] Proia P., Rossi C., Alioto A., Amato A., Polizzotto C., Pagliaro A., Kuliś S., Baldassano S. (2023). MiRNAs Expression Modulates Osteogenesis in Response to Exercise and Nutrition. Genes.

[43] Wan X., Zhang W., Dai L., Chen L. (2024). The Role of Extracellular Vesicles in Bone Regeneration and Associated Bone Diseases. Curr. Issues Mol. Biol..

[44] Holliday L.S., Patel S.S., Rody W.J. (2021). RANKL and RANK in extracellular vesicles: Surprising new players in bone remodeling. Extracell. Vesicles Circ. Nucleic Acids.

[45] Zhu S., Yan M.-Q., Masson A., Chen W., Li Y.-P. (2026). Cell signaling and transcriptional regulation of osteoclast lineage commitment, differentiation, bone resorption and diseases. Cell Discov..

[46] Ansari S., de Wildt B.W.M., Vis M.A.M., de Korte C.E., Ito K., Hofmann S., Yuana Y. (2021). Matrix Vesicles: Role in Bone Mineralization and Potential Use as Therapeutics. Pharmaceuticals.

[47] Wang T., Huang S., He C. (2022). Senescent cells: A therapeutic target for osteoporosis. Cell Prolif..

[48] Zhou P., Zheng T., Zhao B. (2022). Cytokine-mediated immunomodulation of osteoclastogenesis. Bone.

[49] Lombardo M., Aiello G., Fratantonio D., Karav S., Baldelli S. (2024). Functional Role of Extracellular Vesicles in Skeletal Muscle Physiology and Sarcopenia: The Importance of Physical Exercise and Nutrition. Nutrients.

[50] Sandonà M., Consalvi S., Tucciarone L., De Bardi M., Scimeca M., Angelini D.F., Buffa V., D’AMico A., Bertini E.S., Cazzaniga S. (2020). HDAC inhibitors tune miRNAs in extracellular vesicles of dystrophic muscle-resident mesenchymal cells. Embo Rep..

[51] Oliveira G.P., Porto W.F., Palu C.C., Pereira L.M., Petriz B., Almeida J.A., Viana J., Filho N.N.A., Franco O.L., Pereira R.W. (2018). Effects of Acute Aerobic Exercise on Rats Serum Extracellular Vesicles Diameter, Concentration and Small RNAs Content. Front. Physiol..

[52] Alfonzo M.C., Al Saedi A., Fulzele S., Hamrick M.W. (2022). Extracellular Vesicles as Communicators of Senescence in Musculoskeletal Aging. JBMR Plus.

[53] Wang L.-X., Zhang X., Guan L.-J., Pen Y. (2022). Welche Rolle spielen extrazelluläre Vesikel bei der Entwicklung von Frailty und Sarkopenie?. Z. Gerontol. Geriatr..

[54] Gil Izquierdo S., Pilar A.F., Rios J.L., Lim K.S., Toh W.S., Liu C., Gimona M., Gawlitta D., Man K. (2025). Advances in extracellular vesicle-based nanomedicine for regenerative orthopaedics. J. Nanobiotechnology.

[55] Huang X., Lan Y., Shen J., Chen Z., Xie Z. (2022). Extracellular Vesicles in Bone Homeostasis: Emerging Mediators of Osteoimmune Interactions and Promising Therapeutic Targets. Int. J. Biol. Sci..

[56] Biswas S., Gangadaran P., Dhara C., Ghosh S., Phadikar S.D., Chakraborty A., Mahajan A.A., Mondal R., Chattopadhyay D., Banerjee T. (2025). Extracellular Vesicles in Osteogenesis: A Comprehensive Review of Mechanisms and Therapeutic Potential for Bone Regeneration. Curr. Issues Mol. Biol..

[57] Goto K., Watanabe D., Yanagida K., Takagi T., Mizushima A. (2025). Harnessing miRNA-Containing Extracellular Vesicles from Mesenchymal Stromal Cell-Derived Extracellular Vesicles for Regeneration of Bone Defects: A Narrative Review of Mechanisms, Biomaterials, and Clinical Translation. Cancers.

[58] Yuan F.-L., Wu Q.-Y., Miao Z.-N., Xu M.-H., Xu R.-S., Jiang D.-L., Ye J.-X., Chen F.-H., Zhao M.-D., Wang H.-J. (2018). Osteoclast-Derived Extracellular Vesicles: Novel Regulators of Osteoclastogenesis and Osteoclast–Osteoblasts Communication in Bone Remodeling. Front. Physiol..

[59] Al-Sharabi N., Mohamed-Ahmed S., Shanbhag S., Kampleitner C., Elnour R., Yamada S., Rana N., Birkeland E., Tangl S., Gruber R. (2024). Osteogenic human MSC-derived extracellular vesicles regulate MSC activity and osteogenic differentiation and promote bone regeneration in a rat calvarial defect model. Stem Cell Res. Ther..

[60] Carbonare L.D., Minoia A., Braggio M., Piritore F.C., Vareschi A., Cominacini M., Gandini A., Antoniazzi F., Cui D., Romanelli M.G. (2025). Extracellular Vesicles in Osteogenesis: Comparative Analysis of Stem Cell Sources, Conditioning Strategies, and In Vitro Models Toward Advanced Bone Regeneration. Cells.

[61] Morrell A.E., Brown G.N., Robinson S.T., Sattler R.L., Baik A.D., Zhen G., Cao X., Bonewald L.F., Jin W., Kam L.C. (2018). Mechanically induced Ca^2+^ oscillations in osteocytes release extracellular vesicles and enhance bone formation. Bone Res..

[62] Zhou Y.-K., Han C.-S., Zhu Z.-L., Chen P., Wang Y.-M., Lin S., Chen L.-J., Zhuang Z.-M., Zhou Y.-H., Yang R.-L. (2024). M2 exosomes modified by hydrogen sulfide promoted bone regeneration by moesin mediated endocytosis. Bioact. Mater..

[63] Kim J.-M., Lin C., Stavre Z., Greenblatt M.B., Shim J.-H. (2020). Osteoblast-Osteoclast Communication and Bone Homeostasis. Cells.

[64] Wang Y., Wen J., Lu T., Han W., Jiao K., Li H. (2024). Mesenchymal Stem Cell-Derived Extracellular Vesicles in Bone-Related Diseases: Intercellular Communication Messengers and Therapeutic Engineering Protagonists. Int. J. Nanomed..

[65] Zhu S., Yao F., Qiu H., Zhang G., Xu H., Xu J. (2017). Coupling factors and exosomal packaging microRNAs involved in the regulation of bone remodelling. Biol. Rev..

[66] Deng L., Wang Y., Peng Y., Wu Y., Ding Y., Jiang Y., Shen Z., Fu Q. (2015). Osteoblast-derived microvesicles: A novel mechanism for communication between osteoblasts and osteoclasts. Bone.

[67] Mazziotta C., Lanzillotti C., Iaquinta M.R., Taraballi F., Torreggiani E., Rotondo J.C., Otòn-Gonzalez L., Mazzoni E., Frontini F., Bononi I. (2021). MicroRNAs Modulate Signaling Pathways in Osteogenic Differentiation of Mesenchymal Stem Cells. Int. J. Mol. Sci..

[68] Fakhry M., Hamade E., Badran B., Buchet R., Magne D. (2013). Molecular mechanisms of mesenchymal stem cell differentiation towards osteoblasts. World J. Stem Cells.

[69] Golub E.E. (2009). Role of matrix vesicles in biomineralization. Biochim. Biophys. Acta (BBA) -Gen. Subj..

[70] Sheng M.H.-C., Rundle C.H., Lau K.-H.W. (2025). Microvesicles Released by Osteoclastic Cells Exhibited Chondrogenic, Osteogenic, and Anti-Inflammatory Activities: An Evaluation of the Feasibility of Their Use for Treatment of Osteoarthritis in a Mouse Model. Cells.

[71] Tang L., Wang P., Sheng S., Yang H., Jiang Y., Jing Y., Liu H., Su J. (2025). Extracellular vesicles in bone aging: Therapeutic strategies and applications. Extracell. Vesicles Circ. Nucleic Acids.

[72] Zhu Y., Fang X., Zhang S., Liao Y., Lin H., Chen P., Yang M., Huang J., Wang X. (2026). The Dual Role of Extracellular Vesicles in Aging and Age-Related Diseases: Pathophysiology and Therapeutic Potential. Int. J. Nanomed..

[73] Putri P.H.L., Alamudi S.H., Dong X., Fu Y. (2025). Extracellular vesicles in age-related diseases: Disease pathogenesis, intervention, and biomarker. Stem Cell Res. Ther..

[74] Ingangi V., Amoroso D., Passaro E., Di Vaia V., Maltoni F., Narimanfar G., Minopoli M., Rosa R., Ametrano M., Di Gennaro E. (2026). Understanding hairy cell leukemia in the context of mature B−cell neoplasms: Tumor microenvironment and extracellular vesicle contribution to disease pathogenesis. Front. Immunol..

[75] Yao H., Cui Y., Li P., Sun S., Peng C., Wu D. (2026). The role and mechanisms of bone microenvironment regulators in osteoporosis: Novel intervention strategies for addressing the challenges of aging. Front. Endocrinol..

[76] Yuan Y., Guo J., Zhang L., Tong X., Zhang S., Zhou X., Zhang M., Chen X., Lei L., Li H. (2019). MiR-214 Attenuates the Osteogenic Effects of Mechanical Loading on Osteoblasts. Int. J. Sports Med..

[77] Sun W., Zhao C., Li Y., Wang L., Nie G., Peng J., Wang A., Zhang P., Tian W., Li Q. (2016). Osteoclast-derived microRNA-containing exosomes selectively inhibit osteoblast activity. Cell Discov..

[78] Ma Q., Liang M., Wu Y., Ding N., Duan L., Yu T., Bai Y., Kang F., Dong S., Xu J. (2019). Mature osteoclast–derived apoptotic bodies promote osteogenic differentiation via RANKL-mediated reverse signaling. J. Biol. Chem..

[79] Uenaka M., Yamashita E., Kikuta J., Morimoto A., Ao T., Mizuno H., Furuya M., Hasegawa T., Tsukazaki H., Sudo T. (2022). Osteoblast-derived vesicles induce a switch from bone-formation to bone-resorption in vivo. Nat. Commun..

[80] Luo J., Li L., Shi W., Xu K., Shen Y., Dai B. (2025). Oxidative stress and inflammation: Roles in osteoporosis. Front. Immunol..

[81] Stojanovic B., Jovanovic I., Stojanovic M.D., Stojanovic B.S., Kovacevic V., Radosavljevic I., Jovanovic D., Kovacevic M.M., Zornic N., Arsic A.A. (2025). Oxidative Stress-Driven Cellular Senescence: Mechanistic Crosstalk and Therapeutic Horizons. Antioxidants.

[82] Hooten N.N., Byappanahalli A.M., Vannoy M., Omoniyi V., Evans M.K. (2022). Influences of age, race, and sex on extracellular vesicle characteristics. Theranostics.

[83] Tarantino U., Greggi C., Visconti V.V., Cariati I., Bonanni R., Gasperini B., Nardone I., Gasbarra E., Iundusi R. (2022). Sarcopenia and bone health: New acquisitions for a firm liaison. Ther. Adv. Musculoskelet. Dis..

[84] Inoue T., Maeda K., Satake S., Matsui Y., Arai H. (2021). Osteosarcopenia, the co-existence of osteoporosis and sarcopenia, is associated with social frailty in older adults. Aging Clin. Exp. Res..

[85] Kong X., Liu H., Chen S., Liu Z., Chen Q., Li X., Hu H., Su J., Shi Y. (2025). Bioengineered bacterial extracellular vesicles for targeted delivery of an osteoclastogenesis-inhibitory peptide to alleviate osteoporosis. J. Control. Release.

[86] Shi Y., Zheng Z., Li Y., Wang Y., Azevedo H.S., Liu X., Zhang C., Hu H. (2026). Bacterial extracellular vesicles as bioactive nanocarriers for wound treatment. Acta Pharm. Sin. B.

[87] Liu J., Chen C., Liu Z., Luo Z., Rao S., Jin L., Wan T., Yue T., Tan Y., Yin H. (2021). Extracellular Vesicles from Child Gut Microbiota Enter into Bone to Preserve Bone Mass and Strength. Adv. Sci..

[88] Brotto M., Bonewald L. (2015). Bone and muscle: Interactions beyond mechanical. Bone.

[89] He Y., Wang Y., Weng K., Weng X., Yuan Y. (2025). Roles of skeletal muscle-derived exosomes in osteoporosis. J. Transl. Med..

[90] Zhang B., Chen Y., Chen Q., Zhang H. (2025). Exosomal miRNAs in muscle-bone crosstalk: Mechanistic links, exercise modulation and implications for sarcopenia, osteoporosis and osteosarcopenia. Metabolism.

[91] Huang B., Peng Z., Kang L. (2026). The Role of Extracellular Vesicles MicroRNAs in Sarcopenia: From Aging to Multi-Morbidity. Aging Med..

[92] Chen C., Sun M., Wang J., Su L., Lin J., Yan X. (2021). Active cargo loading into extracellular vesicles: Highlights the heterogeneous encapsulation behaviour. J. Extracell. Vesicles.

[93] Liu W., Huang J., Chen F., Xie D., Wang L., Ye C., Zhu Q., Li X., Li X., Yang L. (2021). MSC-derived small extracellular vesicles overexpressing miR-20a promoted the osteointegration of porous titanium alloy by enhancing osteogenesis via targeting BAMBI. Stem Cell Res. Ther..

[94] Liu W., Li L., Rong Y., Qian D., Chen J., Zhou Z., Luo Y., Jiang D., Cheng L., Zhao S. (2020). Hypoxic mesenchymal stem cell-derived exosomes promote bone fracture healing by the transfer of miR-126. Acta Biomater..

[95] Ma Y., Sun L., Zhang J., Chiang C., Pan J., Wang X., Kwak K.J., Li H., Zhao R., Rima X.Y. (2023). Exosomal mRNAs for Angiogenic–Osteogenic Coupled Bone Repair. Adv. Sci..

[96] Pizzicannella J., Gugliandolo A., Orsini T., Fontana A., Ventrella A., Mazzon E., Bramanti P., Diomede F., Trubiani O. (2019). Engineered Extracellular Vesicles From Human Periodontal-Ligament Stem Cells Increase VEGF/VEGFR2 Expression During Bone Regeneration. Front. Physiol..

[97] Du Z., Rizzo S.A., Sarrafian T.L., Bagwell M.S., Mahlberg R.C., Amontree A., Schiebel P., Tauferner D.M., LeBrasseur Z.S., Witt T.A. (2025). Engineered BMP2/BMP7 extracellular vesicles induce autocrine BMP release driving SMAD phosphorylation to promote bone formation. npj Regen. Med..

[98] Li S., Sheng Y., You Y., Wang Y., Zhang Y., Tao J., Wu C., Jiang X. (2025). Study on VEGFA mRNA delivery via GelMA hydrogel-encapsulated extracellular vesicles for enhanced bone regeneration. Mater. Today Bio.

[99] Yu T., Zhao I.S., Pan H., Yang J., Wang H., Deng Y., Zhang Y. (2024). Extracellular vesicle-functionalized bioactive scaffolds for bone regeneration. Asian J. Pharm. Sci..

[100] Ma Y., Dong S., Wu A., Jeong S.D., Lee A.S., Jiang W., Kim B.Y.S. (2026). Engineering challenges and translational opportunities in emerging gene delivery platforms. Nat. Biomed. Eng..

